# Pembrolizumab Dual Endocrine Toxicity in a Metastatic Non-small Cell Lung Cancer Patient

**DOI:** 10.7759/cureus.110075

**Published:** 2026-06-01

**Authors:** Sawsan Al-Lababidi, Rand Alkhlaifat, Maria Herrera-Gonzalez, Darshi Sunderam

**Affiliations:** 1 Internal Medicine, Saint Michael’s Medical Center, Newark, USA; 2 Endocrinology and Diabetes, Saint Michael’s Medical Center, Newark, USA

**Keywords:** central adrenal insufficiency, hypophysitis, immune-related adverse event (irae), pd-4 inhibitor, pembrolizumab, thyroditis

## Abstract

Immune checkpoint inhibitors can cause endocrine toxicities, although involvement of multiple hormonal axes is uncommon. Hypophysitis associated with programmed death-1 inhibitors such as pembrolizumab may lead to life-threatening central adrenal insufficiency and can present diagnostic challenges. We present the case of a 58-year-old woman with metastatic non-small cell lung cancer who developed pembrolizumab-induced hypophysitis resulting in central adrenal insufficiency with delayed recognition, and concurrent immune-related thyroiditis.

## Introduction

Immune checkpoint inhibitors (ICIs), including programmed death-1 (PD-1) inhibitors such as pembrolizumab, have revolutionized cancer therapy but pose diagnostic challenges due to immune-related adverse events (irAEs) that can mimic disease progression or treatment toxicity [1]. ICIs enhance immune-mediated tumor destruction but also affect normal organ tissues, and cause cell death, leading to clinically significant irAEs, such as thyroid dysfunction, hypophysitis, and adrenal insufficiency [1,2].

Immunotherapy-induced endocrinopathies are well documented; however, involvement of multiple endocrine axes in a single patient is rare [3]. Although hypophysitis is less frequent with PD-1 inhibitors than with cytotoxic T-lymphocyte-associated antigen 4 (CTLA-4) inhibitors [4], it can still result in life-threatening adrenal insufficiency. We report a case of pembrolizumab-induced hypophysitis resulting in central adrenal insufficiency with delayed recognition and concurrent immune-related thyroiditis.

## Case presentation

A 58-year-old female with a history of metastatic non-small cell lung cancer (diagnosed in May 2025) presented with progressive fatigue, nausea, vomiting, and diarrhea. Her oncologic history was significant for treatment with carboplatin, paclitaxel, and pembrolizumab every 21 days for four cycles (June 5, 2025, to August 12, 2025), followed by pembrolizumab maintenance therapy every 21 days, initiated on September 2, 2025.

Baseline endocrine evaluation before initiation of pembrolizumab maintenance therapy on September 2, 2025, demonstrated normal adrenal and thyroid function, including a serum cortisol level of 17.78 µg/dL, thyroid-stimulating hormone (TSH) of 2.62 µIU/mL, and free thyroxine (T4) of 1.02 ng/dL.

She presented on September 23, 2025, with one week of fatigue, nausea, vomiting, diarrhea, and poor oral intake. On presentation, she was hypotensive, with blood pressure readings as low as 85/52 mmHg, and remained afebrile with preserved oxygen saturation on room air. Laboratory evaluation revealed acute kidney injury, likely secondary to volume depletion in the setting of gastrointestinal losses, along with mild hyponatremia (Na) of 135 mmol/L. She was treated with intravenous fluids with improvement in renal function and was discharged the following day without endocrine evaluation.

The patient re-presented on September 30, 2025, with worsening fatigue, persistent nausea, vomiting, diarrhea, and generalized weakness. On admission, she remained hypotensive with a blood pressure of 81/50 mmHg and was found to have persistent acute kidney injury. Given the absence of an identifiable infectious source and ongoing gastrointestinal symptoms, ICI-related colitis was suspected. However, colonoscopy revealed no evidence of colitis or other significant abnormalities.

Further evaluation revealed a markedly low serum cortisol level of 1.01 µg/dL, confirmed by a morning cortisol level of 0.64 µg/dL. The adrenocorticotropic hormone (ACTH) level was inappropriately normal at 8.4 pg/mL despite profound hypocortisolemia, consistent with central adrenal insufficiency.

The patient was treated with 100 mg intravenous hydrocortisone, with subsequent improvement in blood pressure and clinical symptoms, followed by a physiologic dose of steroid therapy, 20 mg in the morning and 10 mg in the evening.

Thyroid function tests were notable for a suppressed TSH of 0.024 µIU/mL and elevated T4 of 2.24 ng/dL, consistent with the thyrotoxic phase of ICI-induced thyroiditis.

The patient was diagnosed with ICI-induced hypophysitis resulting in central adrenal insufficiency and thyroiditis. The onset of symptoms occurred approximately three weeks after initiation of pembrolizumab maintenance therapy. She was transitioned to oral hydrocortisone replacement therapy and discharged with outpatient endocrinology and oncology follow-up, with a plan to continue her pembrolizumab maintenance therapy for two years.

Subsequent laboratory evaluation in the endocrine clinic demonstrated progression from thyrotoxicosis to hypothyroidism, with elevated TSH (40 mIU/L) and low free T4 (0.32 ng/dL), consistent with the expected biphasic course of immune-related thyroiditis.

Magnetic resonance imaging (MRI) of the brain performed as an outpatient in October 2025 demonstrated no evidence of pituitary enlargement or adenoma, suggesting that imaging may be normal in ICI-induced hypophysitis. A summary of key laboratory trends during the clinical course is presented in Table 1.

**Table 1:** Key laboratory trends during clinical course. Arrows indicate relative change from baseline. ACTH level was inappropriately normal in the setting of severe cortisol deficiency, supporting central adrenal insufficiency. The elevated follow-up cortisol level is attributable to exogenous glucocorticoid therapy. ACTH = adrenocorticotropic hormone; TSH = thyroid-stimulating hormone; T4 = free thyroxine

Parameter	Reference range	Baseline (9/2/2025)	First admission (9/23/2025)	Second admission (9/30/2025)	Follow-up (11/18/2025)
Cortisol (µg/dL)	2–25	17.78	—	1.01 ↓	25.61 ↑*
ACTH (pg/mL)	7.2–63.3	—	—	8.4	—
TSH (µIU/mL)	0.550–4.780	2.62	—	0.024 ↓	40.04 ↑
Free T4 (ng/dL)	0.8–1.8	1.02	—	2.24 ↑	0.32 ↓
Sodium (mmol/L)	136–145	—	135	135	—
Creatinine (mg/dL)	0.6–1.2	0.86	2.1 ↑	4.13 ↑	0.82

## Discussion

The patient initially presented with hypotension, fatigue, nausea, vomiting, and diarrhea. These non-specific symptoms can resemble those of more common conditions, such as dehydration, infection, or chemotherapy-related adverse effects. At her first emergency department visit, she was treated for presumed volume depletion without an endocrine evaluation, despite documented hypotension and ongoing pembrolizumab therapy. Upon re-presentation, she was admitted and underwent a comprehensive endocrine evaluation. Central adrenal insufficiency associated with pembrolizumab therapy was indicated by markedly low cortisol levels and an inappropriately normal ACTH concentration.

In the context of severe cortisol deficiency, ACTH is expected to be elevated; therefore, a normal ACTH level is physiologically inadequate and indicates central adrenal insufficiency. Hypophysitis, which presented in our case as central adrenal insufficiency, is significantly more common with CTLA-4 inhibitors, occurring in up to 10% of treated patients, whereas PD-1 inhibitor-induced hypophysitis is exceedingly rare, with an incidence of less than 1% [4]. Despite its lower frequency, PD-1 inhibitor-associated hypophysitis can result in life-threatening adrenal insufficiency if not recognized and treated promptly. This emphasizes the need to maintain a high index of suspicion for adrenal insufficiency in patients receiving PD-1 inhibitors, particularly when they present with unexplained hypotension and constitutional symptoms.

An additional feature of this case is the coexistence of thyroid dysfunction. The patient initially showed biochemical thyrotoxicosis, which progressed to hypothyroidism on follow-up, consistent with the biphasic course of ICI-induced thyroiditis [5]. Thyroiditis and hypophysitis are well-recognized endocrine irAEs; however, reports of their concurrent occurrence in the same patient remain limited [3]. Therefore, a comprehensive hormonal evaluation is required in patients receiving immunotherapy.

The temporal relationship between the initiation of pembrolizumab maintenance therapy and the onset of symptoms further supports an immune-mediated etiology. Endocrine irAEs may occur at variable time points during treatment but frequently develop within weeks to months after therapy initiation [1]. Notably, this patient had normal baseline cortisol and thyroid function before starting pembrolizumab, which strengthens the causal association.

Outpatient follow-up MRI of the pituitary gland was unremarkable, consistent with prior reports indicating that PD-1 inhibitor-associated hypophysitis may occur without radiographic abnormalities [6]. While pituitary enlargement is observed in most cases of CTLA-4 inhibitor-associated hypophysitis, MRI abnormalities are seen in only a minority of patients treated with PD-1 inhibitors alone (approximately 28%) [4]. Therefore, the diagnosis is primarily based on biochemical and clinical findings rather than radiologic evidence.

## Conclusions

Hypophysitis resulting in central adrenal insufficiency is an important and potentially life-threatening complication of ICIs, including pembrolizumab, and should be suspected in patients presenting with hypotension, fatigue, or gastrointestinal symptoms. Furthermore, ICI-related endocrinopathies can affect multiple hormonal axes, either simultaneously or sequentially, necessitating comprehensive hormonal evaluation and close follow-up. Early recognition and prompt treatment are essential to prevent life-threatening complications and require a high index of suspicion for endocrine toxicities in patients receiving immunotherapy.
